# Utilization of Coniferous and Deciduous Tree and Paper Ashes as Fillers of Rigid Polyurethane/Polyisocyanurate (PU/PIR) Foams

**DOI:** 10.3390/ma18051165

**Published:** 2025-03-05

**Authors:** Joanna Liszkowska, Magdalena Stepczyńska, Andrzej Trafarski, Justyna Miłek, Tomasz Karasiewicz

**Affiliations:** 1 Department of Chemistry and Technology of Polyurethanes, Faculty of Materials Engineering, Kazimierz Wielki University, J. K. Chodkiewicza 30, PL 85-064 Bydgoszcz, Poland; 2 Department of Polymer Materials Engineering, Faculty of Materials Engineering, Kazimierz Wielki University, J. K. Chodkiewicza 30, PL 85-064 Bydgoszcz, Poland; 3 Department of Construction Materials and Biomaterials, Faculty of Materials Engineering, Kazimierz Wielki University, J. K. Chodkiewicza 30, PL 85-064 Bydgoszcz, Poland; 4 Department of Chemical and Bioprocess Engineering, Faculty of Chemical Technology and Engineering, Bydgoszcz University of Technology, Seminaryjna 3, PL 85-326 Bydgoszcz, Poland

**Keywords:** rigid foam, DSC, EDS, FTIR, thermal properties, oak ash, birch, pine, spruce, deciduous tree

## Abstract

Five series of rigid polyurethane–polyisocyanurate (RPU/PIR) foams were obtained. They were modified by ashes from burning paper (P) and wood: conifers (pine—S, spruce—S’) and deciduous trees (oak—D, birch—B). The ash was added to rigid polyurethane–polyisocyanurate foams (PU/PIR). In this way, five series of foams with different ash contents (from 1 to 9% wt.) were obtained: PP, PS, PD, PS’, PB. The model foam (reference—W) was obtained without filler. The basic properties, physico-mechanical, and thermal properties of the ashes and obtained foams were examined. It was specified, among other things, the cellular structure by scanning electron microscopy (SEM), and changes in chemical structure by Fourier-transform infrared spectroscopy (FTIR) were compared. The obtained foams were also subjected to thermostating in a circulating air dryer in increased temperature (120 °C) for 48 h. Ash tests showed that their skeletal density is about 2.9 g/cm^3^, and the pH of their solutions ranges from 9 to 13. The varied color of the ashes affected the color of the foams. SEM-EDS tests showed the presence of magnesium, calcium, silicon, potassium, aluminum, phosphorus, sodium, and sulfur in the ashes. Foam tests showed that pine ash is the most beneficial for foams, because it increases their compressive strength three times compared to W foam and improves their thermal stability. All ashes cause the residue after combustion of the foams (retention) to increase and the range of combustion of the samples to decrease.

## 1. Introduction

Combustion by-products (CBPs) are created during the combustion of hard coal and brown coal in Combined Heat and Power (CHP) plants and power plants [1,2]. In the years 1990–2009, coal extraction and consumption systematically increased [3,4]. In 1990, global hard coal extraction amounted to 3497 Mt and in 2009 to 5924 Mt [5,6]. Burning of coal may have been more than ten billion tons per year in 2023 [4]. The current energy policy towards the natural environment, apart from climate devastation, results in an increasing area of landfills. Every year, areas of about 1.2–1.4 km^2^ are designated for them in Poland (1999) [7], which exposes soil and groundwater to contamination with heavy metals. The increase in coal consumption causes landfills to fill up with CBP waste [3]. This generates costs of waste management, e.g., related to the acquisition of appropriate land [8]. Ashes released into the atmosphere constitute an ecological problem. The most serious threat is the phenomenon of secondary dusting. The waste is carried by the wind, causing air pollution, and then falls to the surface of the area around the landfill. Here, it is leached by atmospheric precipitation, surface water, or groundwater. Another threat is the risk of landslides of deposited waste from above-level landfills. Locating fly ash landfills on the surface is a necessary evil [9].

In order to reduce the amount of fly ash waste, it is processed [8]. Thanks to this, it is used in many industries. The presence of minerals in ash waste means that it is used in agriculture or horticulture [4] as a soil binder. It is also used in construction (so-called civil engineering) [8,10,11], e.g., concretes with ash are used in the construction of skyscrapers in Dubai [8], road construction, ceramics [12], foundry [7], metallurgical industry [12], mining [13], and in the construction of sedimentation ponds [14]. In the literature, one can also find information on ashes generated from the combustion of biomass with coal, described as proecological [15].

The use of waste from ash requires testing its properties. Recognizing the properties of ash facilitates the selection of the appropriate method of its management and use. Ashes must meet the normative requirements, depending on their use [16,17,18,19,20,21,22]. Thanks to the classification of ash into a given group, its physicochemical properties are also known, which facilitates its economic use [9]. The properties of ash also depend on, among others, combustion conditions, e.g., in the laboratory [12] or during uncontrolled combustion in stoves or fireplaces. The moisture content of the burned fuel (coal, wood, paper) is also important in terms of the properties of the resulting ash. More soot (coal) is produced from wet wood. Waste is also produced in households. Here, we are dealing not only with coal ash. Using paper as a kindling for burning in fireplaces or free-standing stoves or central heating stoves, we generate waste in the form of ash in a huge amount in relation to the material used. Fireplaces are increasingly being built in homes. Most often, birch and oak wood is burned in fireplaces, less often pine or spruce. In central heating (CO) stoves, pellets (granulates) made of wood are often burned. Pellets, just like split wood, also generate large amounts of ash, the more the poorer the quality of the pellet burned. Biomass made of wood is also used [23].

The scope of this research includes the management of waste from various species of deciduous and coniferous trees and from paper as a filler for polyurethane–polyisocyanurate foams (PU/PIR). Polyurethane foams (PUs) are becoming increasingly popular, especially in construction. Their thermal insulation properties and resistance to external factors have been the subject of scientific research for many years [24,25,26].

In the production of PU, an important aspect is the reduction of the cost of the finished product. For this purpose, PU is modified at the stage of production of raw materials for it (e.g., modification of polyols by adding fillers) [27,28]. An important aspect here is also environmental protection [29]. With environmental protection in mind, the possibilities of waste management are in the form of ash from the combustion of various types of solid fuels used: two species of deciduous trees, two coniferous trees, and paper. The literature shows that research on ash mainly concerns ash (fly) from coal combustion, called microspheres [30]. Kuznia et al. [31] added microspheres to polyols during the synthesis of PU foam. It was shown that foams modified in this way have better properties, e.g., heat, thermal (greater thermal stability), and greater fire resistance. This is mainly due to the presence of gas in the microspheres [32]. Paper waste was also used as a modifier of PU foams [33,34]. The modifier decreased the thermal conductivity and compressive strength and increased water vapor resistance and density. The studies showed that fly ash caused a decrease in the dimensional stability of the obtained product [34].

The aim of this research is to use ashes from various species of coniferous trees (pine, spruce), deciduous trees (oak, birch), and from paper (cardboard) as a modifier of rigid PU/PIR foams.

As it results from the analyzed literature, so far, the research has mainly been conducted on fly ash and its application in construction and road construction.

This article examined the properties of solid ash obtained as a result of free, uncontrolled combustion in a fireplace of various species of dry deciduous trees (pine—S, oak—D), coniferous trees (spruce—S’, birch—B), and paper from cardboard boxes (paper—P). The ash was added to rigid polyurethane–polyisocyanurate (PU/PIR) foams, thus obtaining five series of foams with different ash content (from 1 wt. % to 9 wt.%): PS, PD, PS’, PB, PP.

These fillers are designed to, among other things, increase the physical strength of the foams, temperature resistance, and thermal stability, and reduce flammability. The use of ash for foams meant that they were still a lightweight material but with improved utility properties. The use of ashes for polyurethanes perfectly fits into the program of reducing the toxic impact of conventional energy on the natural environment and has become particularly important in the face of the constantly growing consumption of wood and paper [35,36].

## 2. Materials and Methods

### 2.1. Materials

The following raw materials were used to obtain the polyurethane material:

- Polyether polyol (polyoxyalkylenetriol) (ASTM D 2849-69), Rokopol G 441, based on glycerin, with antioxidants, without BHT–butylhydroxytoluene, with hydroxyl number of 330–360 mgKOH/g, about dynamic viscosity at 25 °C (in 250–310 mPas), (PCC Rokita S.A., Brzeg Dolny, Poland);
- Diisocyanate Purocyn B (ASTM D 1638–70) (Purinova, Bydgoszcz, Poland), main component 4,4’-diphenyl-methane-diisocyanate (MDI), viscosity 200 mPas, density1.23 g/cm^3^ (at 25 °C), 31.0%-NCO groups;
- Catalytic system: (a) 33% solution of anhydrous potassium acetate in diethylene glycol (both Chempur, Piekary Śląskie, Poland), (b) 33% solution of DABCO (1.4-diazabicyclo[2.2.2]octane, Alfa Aesar, Haverhill, MA, USA) in diethylene glycol, trimerization polyurethane bond catalyst;
- Structure stabilizer Genapol X 080–a fatty alcohol polyglycol ether (Clariant Produkte, Deutschland, GmbH, Frankfurt am Main, Germany);
- Blowing agent carbon dioxide (in situ in the reaction between water and isocyanate groups);
- Flame retardant Roflam F5–phenol isopropylated phosphate, density 1.15–1.25 g/cm^3^ (in 25 °C), dynamic viscosity 48–67 mPas (25 °C) (PCC Rokita S.A., Brzeg Dolny, Poland) [37].

**Figure 1:**
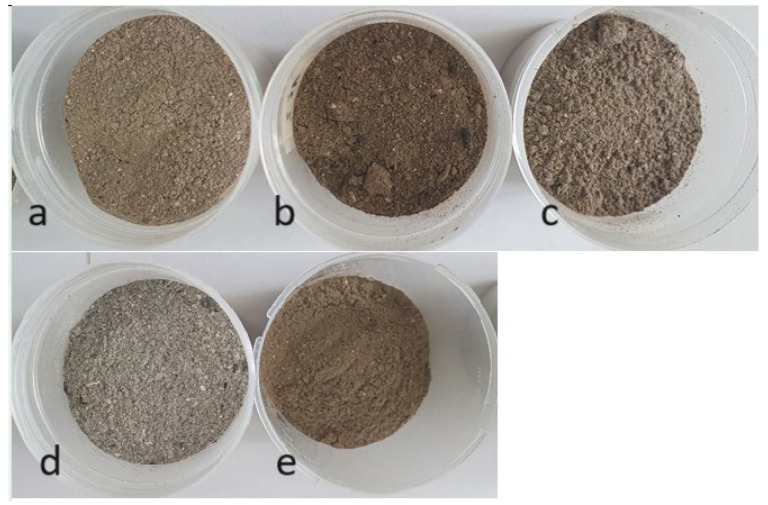
Ashes used for foams: (**a**) pine, (**b**) spruce, (**c**) birch, (**d**) paper, (**e**) oak.

Ashes burned in a home fireplace were used as modifiers of foam properties: S—pine (Figure 1a), S’—spruce (Figure 1b), B—birch (Figure 1c), D—oak (Figure 1e), and P—from cardboard–paper ash (Figure 1d). The wood came from the forest near a town named 86-150 Tlen, located in the Tuchola Forest (Poland), and the cardboard boxes were from one of the supermarkets.

The combustion temperatures of the trees and cardboard were not measured. After each burning of about 200 kg of a given type of material, the fireplace was thoroughly cleaned. When the wood was lit, the kindling was lowered below the fire so that nothing was left.

### 2.2. Preparation of PU/PIR Foam Samples

The calculations of the foam composition were made according to sources [27,38] and calculations provided in [39,40,41]—Table 1—from the two-component system (one-step method, laboratory scale).

Excess polyisocyanate (3.7 R instead of 3.0 R) was used for the reaction with water (0.7R) [42]. The R chemical equivalent (of the NCO group) was calculated according to Equation (1):

R_NCO = 4200/(31%NCO)(1)

where % NCO—content of NCO group in polyisocyanate (%).

The chemical equivalent R (of the hydroxyl group) was calculated according to Equation (2):

R_OH = 56100/HN(2)

where HN—hydroxyl number of polyol (mg KOH/g).

Purocyn B (250.70 g) was component B. Component A consisted of all the remaining raw materials mixed together, i.e., Rokopol G-441 (79.20 g), trimerization catalyst (8.20 g), polyurethane bond catalyst (3.30 g), flame retardant (49.40 g), surfactant (5.60 g), and distilled water (3.15 g). Reference foam W was obtained from components A and B, which were mixed using a mechanical stirrer (1800 rpm, 10 s). The mixture was poured into a sheet metal mold (190 mm × 190 mm × 230 mm). To obtain modified foams, ash fillers were added to foam W, the amount of which was calculated in relation to the sum of the masses of polyol and polyisocyanate.

Foam formulations (Table 1) contained 1–9%wt. of the following ashes: pine—PS or oak—PD or spruce—PŚ or birch—PB or paper—PP. Adding more fillers was not considered because it made mixing the ingredients more difficult.

### 2.3. Methods of Ashes

The analysis of the fly ash parameters (chemical composition, phase composition, textural properties, functional properties), on the basis of which the assessment of their potential use was made, was performed based on the results of tests of physicochemical properties using various research techniques.

#### 2.3.1. Ash Skeletal Density

The analyses were performed using the AccuPyc helium gas pycnometer manufactured by Micromeritics (Norcross, GA, USA). The measurements were performed using the FoamPyc Standard software for measuring the volume of materials and determining the specific density of materials in a helium atmosphere with a purity of 99.999%. The tests were performed on 10 samples placed in a chamber insert (chamber) with a volume of 10 cm^3^. The obtained ashes of a non-homogeneous nature were weighed and subjected to a skeletal density test.

#### 2.3.2. Bulk Density of Ash

The actual density according to PN-EN 1097–3:2000 was tested in a 25 cm^3^ cylinder. The samples were weighed on a laboratory scale with an accuracy of 0.001 g.

#### 2.3.3. Shape and Diameter of Grains by SEM Method

The studies were performed using a scanning electron microscope, “Phenom XL” version 6.4.1, Thermo Fisher Scientific, Eindhoven, The Netherlands (magnification 200×, electron source CeB6, MSA software version 3.8.6.0). The samples were sputtered with gold for 40 s. A specialized program for measuring cells in the SEM method allowed the measurement of ashes’ cell height (H) and width (W).

#### 2.3.4. SEM-EDS

**Figure 2:**
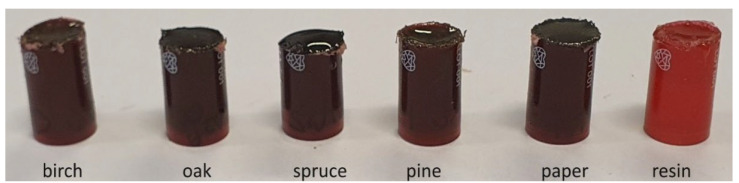
Ashes in resin for SEM-EDS.

The surface topography and composition of ashes was measured using scanning electron microscopy (SEM, p. 2.3.3) equipped with Energy Dispersive Spectroscopy (EDS). Each study was conducted for 30 s; however, only the results from the first 10 s are presented because the results did not indicate the presence of any element after that. Since the ash samples were not suitable for direct testing due to the vacuum generated and the possibility of contaminating the device, the ash was immersed in resin (Figure 2). For this purpose, Epidian 5 epoxy resin was prepared in a hardener in a ratio of 10:1. Then, 1 g of ash was weighed in 30 cm^3^ glass bottles and poured into each 5 g of resin. After thoroughly mixing each ash sample in resin, they were then poured into small plastic containers (Figure 2) with a bottom diameter of 0.9 mm. A test was also performed for the resin itself. After the samples had hardened (two days), each container was ground to a height of 10 mm.

#### 2.3.5. Ash pH

The pH of the ash was measured in aqueous solutions. A sample of each ash weighing 1 g was mixed with 2 g of water. The pH of each solution was measured with a VOLTCRAFT PH-100 ATC pH meter (Toledo).

#### 2.3.6. Ash Solubility

The solubility of ash in water and in the Rokopol RF-551 (polyol used for foam synthesis) was tested.

#### 2.3.7. Measurement of Ashes and Foams Color

The color measurement was performed using the SF80 spectrophotometer (TRI-COLOR sp. z o.o., ul. Jodłowa 50, 32–095 Narama, Poland). The L*, a*, b* values of three samples of each type were measured. The results were averaged. L*, a*, b* are values describing the color trajectory in the CIELAB color space [43]. With these data, ∆E (the difference between two colors in space) was calculated—Equation (3):

(3) $$∆E=\sqrt{{\left(∆L*\right)}^{2}+{\left(∆a*\right)}^{2}+{\left(∆b*\right)}^{2}}$$

where

a*—axis of the coordinate the amount of red (positive “a*” values), the amount of green (negative “a*” values);
b*—axis expressing the amount of yellow color (positive values) or blue (negative values) in color;
L*—the vertical axis of the coordinate system defining the brightness.

#### 2.3.8. DSC of Ashes and Foams

For the examination of changes in foams under heat, this study used a differential scanning calorimeter DSC Q200 (TA Instruments in Advanced Tzero technology). The apparatus works in temperatures −90 to +725 °C (examinations in temperature 0 to 400 °C).

#### 2.3.9. Thermal Analysis (TG, DTG)

The Q500 thermobalance (TA Instruments, New Castle, DE, USA, sample mass of about 21 mg, temperature range 0–1000 °C, temperature change rate 10 °C/min, nitrogen atmosphere) was used for the tests. The TG curves were used to read the temperature values T 5%, T 10%, T 20%, and T 50%, which correspond to the loss of 5%, 10%, 20%, and 5 % of the initial mass of ash or foam, respectively. The DTG curve (first derivative of the TG curve) was used to determine the T_max_ values (temperature of the fastest mass loss).

### 2.4. Methods of Foams

After receiving the foam, it was aged at room temperature for 24 h. Then, the samples were cut on a circular saw with an accuracy of 0.1 mm.

#### 2.4.1. Foaming Process

During the synthesis of foams, the maximum reaction temperature (T_max_) was measured. For this purpose, an electronic thermometer (Browin 145709) placed in the center of each foam block was used.

The maximum reaction temperature (T_max_) in the foams was measured during the synthesis of them, using an electronic thermometer (Browin 145709) placed in the center of the obtained PU/PIR foams.

The foaming process time was controlled using an electronic stopwatch. This was done in accordance with the ASTM D7487 13e1 standard [44].

The foaming times of the following foams were determined in this way:

- creaming—from the start of mixing components A and B until small bubbles appear;
- free rise—from the start of mixing components A and B until the foam stops expanding;
- string gel time—from the start of mixing components A and B until the long strings of sticky material can be pulled away from the foam surface when the surface is touched with a spatula;
- tack free time—from the start of mixing components A and B until the foam surface can be touched with a spatula without sticking.

#### 2.4.2. Accelerated Aging Test

Cubic foam samples with a side of 50 mm were thermostated in a dryer with forced air circulation (at 120 °C for 48 h).

In this way, aging tests were performed (according to ISO 1923:1981 and PN-EN ISO 4590:2016-11, Equations (4)–(7)), i.e.,

- Δlz/Δlp—change in linear dimension in line with/against the direction of foam growth
- ΔV—change in geometric volume
- Δm—loss of mass

Δl_z_ = [(l_z_ − l_0_)/l_0_]∙100% (4)

Δl_p_ = [(l_p_ − l_0_)/l_0_]∙100% (5)

where l_0_—length of the sample before thermostating, according to the direction of foam free rise (mm); l_z_—length of the sample after thermostating, according to the direction of foam rise (mm); and l_p_—length of the sample after thermostating, gains of the direction of foam rise (mm).

ΔV = [(V − V_0_)/V_0_]∙100% (6)

where V_0_—geometrical volume of the sample before thermostating (mm^3^), and V—geometrical volume of the sample after thermostating (mm^3^).

Δm = [(m − m_0_)/m_0_]∙100% (7)

where m_0_—mass of the sample before thermostating (g), and m—mass of the sample after thermostating (g).

#### 2.4.3. Apparent Density

The apparent density of the foams was determined, according to the standard ISO 845:2006, as the ratio of foam mass to its geometrical volume. Cubic samples with a side of 50 mm were used.

#### 2.4.4. Compressive Strength

The Instron universal strength 5544 testing machine was used for this study (according to PN-93/C-89071; ISO 844:2014). Cubic samples with a side of 50 +/− 1 mm were subjected to compression tests at a strain of 10%.

#### 2.4.5. Chemical Structure (FTIR)

Nicolet iS10 (Thermo Fisher Scientific, Waltham, MA, USA) was used to investigate the chemical structure of the foams based on FTIR spectra. The spectroscopic range was 4000–400 cm^−1^, maximum resolution of capability < 0.4 cm^−1^, detector DTGS (with Deuterated alanine doped Tri-Glycine Sulphate).

#### 2.4.6. Flammability

One of the methods of assessing flammability was the retention test (remainder after combustion of foam using the Butler chimney test method) according to the ASTM D3014-73 standard. This is a vertical column measuring 300 × 57 × 54 (mm) (three sheet metal walls and one movable glass column). Five samples of each type (size 150 × 19 × 19 (mm)) were placed in it, previously weighed (accurate to 0.001 g). Then, after installing the glass pane, the samples were burned for 10 s in a propane–butane burner flame. The burner was then moved away, and the sample’s free burning time and retention (combustion residue) were measured with a stopwatch in the vertical test. Retention was calculated according to Equation (8):

(8) $$R=\frac{m}{m0}\times 100\%$$

where

R—retention;
m_0_—mass of the sample before combustion [g];
m—mass of the sample after combustion [g].

Flammability assessment using the horizontal test was performed according to the PN-78 C-05012 standard. The flame range was tested in the horizontal test. For this purpose, a sample measuring 150 × 50 × 13 (mm) was used, placed in a horizontal position and exposed to a flame (propane–butane burner) from one end. This method consists in determining the speed of superficial flame spread on a foam sample. This is the speed at which the flame front moves on the surface of the tested flammable material sample. The test consists in placing a foam sample on a grid mounted horizontally and applying a burning burner to one of the foam ends for 60 s.

#### 2.4.7. Absorbability and Water Absorption

The tests were performed according to ISO 280 2896:2001, which is applicable to rigid porous plastics that do not react with or dissolve in it. Samples (dimensions 150 × 150 × 25 (mm)) were weighed (to 0.001 g), immersed in water for 24 h, and weighed again. The absorbability (N) and water absorption (Ch) values were calculated from Equations (9) and (10).

(9) $$N=\frac{mA-mD}{mD}\times 100\%$$

where mA—mass of the sample after immersion in distilled water (g), and mD—mass of the dry sample (g).

(10) $$\mathrm{Ch}=\frac{mWA-mD}{mD}\times 100\%$$

where mWA—mass of the sample after surface drying (g).

#### 2.4.8. Closed-Cell Content

The analyses were performed using an AccuPyc gas pycnometer manufactured by Micromeritics (USA). The tests were performed in helium. The foams were subjected to analysis determining the number of closed and open cells. Before analysis, the samples were cut in accordance with PN-EN ISO 4590 and ASTM D6226-15. The measurements were performed using FoamPyc software for measuring open and closed cells and testing the compressibility and rupture of cells in foam materials in a nitrogen atmosphere of 99.999% purity, at a temperature of about 25 °C and a pressure of 7 psi (483 hPa). The results are the average of tests on 5 cube samples with a side of 2.5 cm.

#### 2.4.9. Standard Deviation

According to Formula (11), the standard deviation from the arithmetic mean (σ) was calculated.

(11) $$\sigma =\frac{\sqrt{{\left(x1-X\right)}^{2}+{\left(x2-X\right)}^{2}+\ldots +{\left(\mathrm{xn}-X\right)}^{2}}}{n}$$

where x_1_, x_2_,…x_n_—property value data, and X—the arithmetic mean of the x_1_,…x_n_ values

### 2.5. Results and Discussion of Ashes

#### 2.5.1. Bulk and Skeletal Density of Ash

The literature states that the bulk density of loosely poured fresh coal ash waste is about 0.45 g/cm^3^, and the values of the specific density are subject to some fluctuations depending on the samples taken and range from 2.1 to 2.5 g/cm^3^ [45].

The obtained skeletal density results of the ashes tested here did not show significant differences between the samples and amounted to about 2.9 g/cm^3^ (Table 2). The lowest true density was for ash from paper (0.1472 g/cm^3^), the highest for ash from oak (0.3133 g/cm^3^).

#### 2.5.2. SEM of Ashes

Ash grains can be spherical or irregular in shape, empty or solid [46]. Microscopic examinations allow stating that the ashes consist mainly of crumbs and conglomerates of irregular shape and structure, dark in color. Light grains, formed from sand or clay admixtures of coal, occur in smaller quantities [47]. In gravel and sand fractions, crumbs and conglomerates of irregular structure and shape predominate. Rounded grains of regular structure occur in dust and clay fractions. In furnace waste, spherical grains (microspheres) occur; empty inside with a tight coating; glassy, milky or colorless. They are of particular importance for environmental protection. Their bulk density (lower than the density of water) causes them to form a layer that dries easily and is carried away by the wind [36,44].

**Figure 3:**
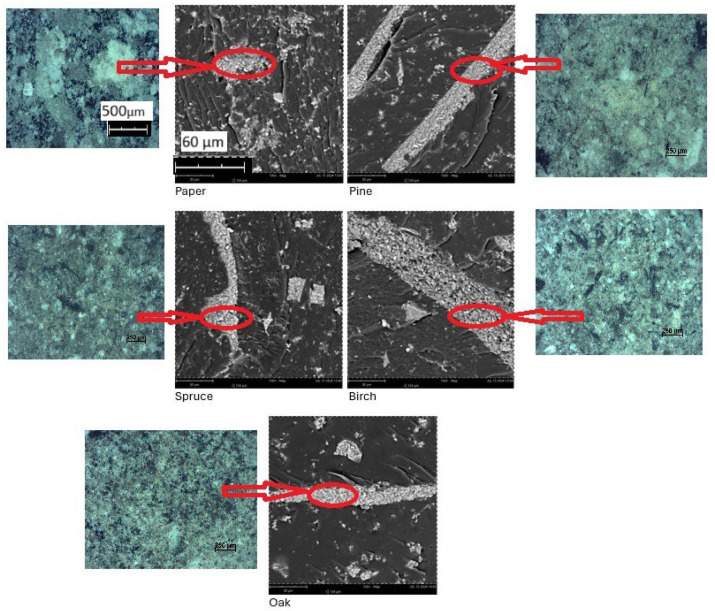
SEM images of ashes.

As a result of SEM examination of the ashes embedded in resin Epidian 5, it was determined that they have the form of light lumps of irregular shapes (Figure 3). Additional photos of the ashes were taken using a 250 μm electron microscope to show the ashes in close-up.

#### 2.5.3. pH of Ashes

The pH value of ashes, according to Fengy et al. [48] and Pandey et al. [49], ranges from 4.5 to 12. Ashes from deciduous trees (poplar, beech, oak) are characterized by high pH values (in the range of 12–13); for coniferous trees, the pH is about 10.

**Figure 4:**
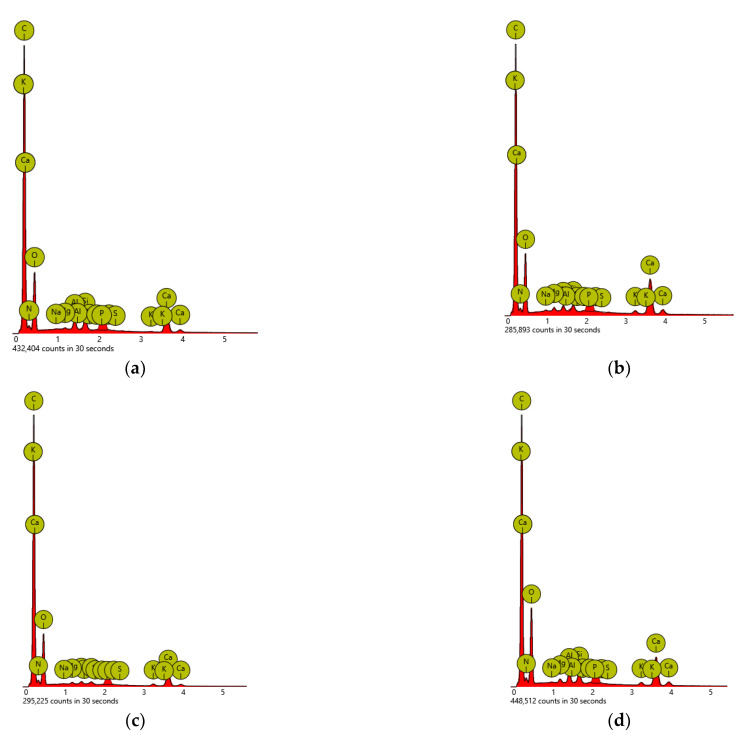

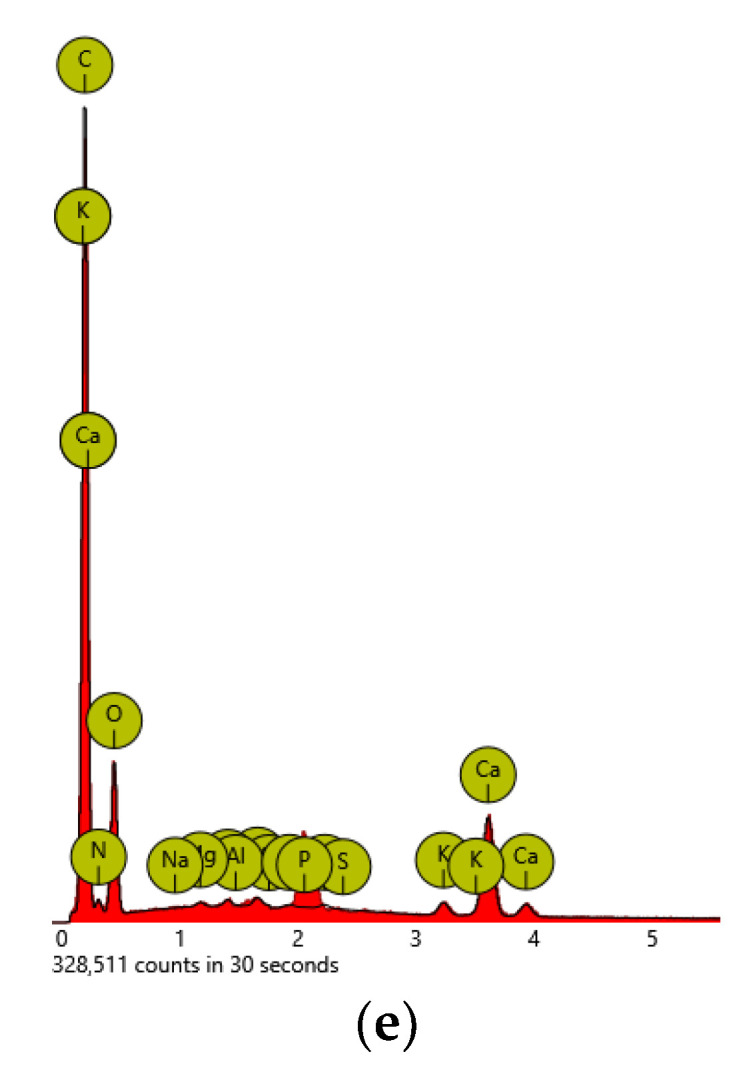
DSC–EDS of ashes: (**a**) paper—P; (**b**) pine—S; (**c**) spruce—S’; (**d**) birch—B; (**e**) oak—D.

The pH values of the ashes tested here ranged from 9 to 13 (Table 2). The highest pH was characteristic of oak ash (13), which would suggest the highest content of metals in it. However, the studies have shown (Table 3) that the most metals were contained in birch ash (pH = 11). The lowest pH (9) was found in paper ash, which contained the smallest amount of metals (Figure 4a). These differences may result from the content of metals and nonmetals, which, in aqueous solutions of the ashes, could form metal and nonmetal oxides in varying proportions. These oxides can react with each other. In this way, they could affect the pH of solutions of ash in water. Amphoteric aluminum also occurs in solutions, which in aqueous solution yields oxide. Aluminum oxide can react with metal to form salts and with nonmetal oxide to form salts as well. This series of reactions could affect the pH of aqueous solutions of ash.

#### 2.5.4. Ash Solubility

Research conducted at the University of Opole [50] has shown that fly ash is susceptible to the release of soluble components. As a result, it shows certain negative trends in terms of its impact on the environment. When examining the solubility of domestic fly ash in water, it was found that, depending on the type of ash, from 0.5% (alumina ash) to 10% or more of its mass (calcium ash) can be dissolved [51,52]. Studies conducted by scientists from the University of Michigan (intense shaking of 100 g of ash in 1 l of distilled water continuously for 24 h) showed that the amount of dissolved solids ranged from 1.0 to 1.7% [52,53]. In natural conditions, during a single contact of ash with water, 0.5 to 1% of the dry mass of ash passed into it. Only as a result of repeated contact of water with ash (in closed circuits) did their leaching increase, i.e., 1 to 1.5% of the dry mass of waste. It was observed that with the increase in the number of times the ash was washed with water, the amount of compounds leached from it decreased [36]. The main substances soluble in water were calcium, magnesium, potassium, sodium hydroxides, and sulphates (98% of all components). Water extracts from ash were characterized by increased alkalinity and hardness. They contained SO_4_^2−^ ions. Trace elements were present in ashes in negligible amounts (0.1–0.3% of the total mass of combustion waste). Trace elements were difficult to wash out; boron and cadmium were the easiest to dissolve. The minimal solubility of heavy metals was caused by the strongly alkaline reaction of water extracts from ash (pH = 9.0–12.0). The exception was chromium, whose solubility increased with increasing alkaline reaction [36,52,53].

The solubility of ash in water and in Rokopol RF-551 polyol used for foam synthesis was tested. The ash formed a suspension with both water and polyol. The dissolved substance’s content was not tested.

#### 2.5.5. SEM-EDS of Ashes

The literature indicates that fly ash contains elements such as oxygen, silicon, aluminum, iron, calcium, magnesium, sodium, potassium, and titanium, whose share ranges from several to several dozen percent, as well as trace elements [54]. Ash mineral components were conventionally expressed as oxides [12]. Diamond et al. [55] determined the basic conventional oxide composition of fly ash: SiO_2_, Al_2_O_3_, Fe_2_O_3_, CaO, MgO, Na_2_O, K_2_O, TiO_2_, SO_3_. In addition to the above-mentioned oxides, iron oxides were also present in the fly ash composition. In addition, P_2_O_5_ and BaO and others were also found, in shares not exceeding several wt. % [54]. Trace amounts of chlorine were also found.

Depending on the mineral content, silicate, aluminum, and calcium ashes were distinguished [56,57,58]. Calcium ashes were divided into two groups depending on the content of calcium oxide, CaO. Low-calcium ashes consisted mainly of aluminosilicate glass. High-calcium ashes contained quartz, free CaO, anhydrite, tricalcium aluminate, and tetracalcium sulphate-aluminate. These components reacted with water and provided the ashes with binding properties [59]. Too high a content of free CaO or MgO meant that such ashes were not very popular among producers of building materials [60,61]. Studies of fly ash from brown coal in the Belchatów district showed a calcium type with a varied chemical composition [62,63,64,65].

Our research results showed that the composition of ash depended on the type of burned raw material (wood, paper) and the species of tree—Figure 4a–e.

DSC-EDS studies showed that the ashes contained the most calcium, Ca, among all metals (Table 3). The highest Ca content was in pine ash (7.13 wt.%) and oak ash (7.27 wt.%). The highest content of silicon, Si, was in paper (1.01 wt.%) and in birch ash (1 wt.%), and the highest content of potassium, K, was in oak ash (0.84 wt.%). The highest content of magnesium, Mg, was in pine ash (0.34 wt.%). The highest content of aluminum, Al, was in pine and birch ash (0.98 wt.% each). The high content of metals in birch ash increased its density (Table 2).

#### 2.5.6. Color of Ashes

The color of raw materials is of great importance in foam synthesis because it affects the color of the foams. The color of the foams is very important for foam recipients. The results of the measured ash color are included in Table 4. The ash color affected the color of the foams to which these ash were added. L determines the lightness, e.g., L* = 0 the darkest, and L* = 100 the lightest. Color channels a* positive determined a lot of red, a* negative determined a lot of green, b* positive determined the amount of yellow, and b* negative determined the amount of blue. For a* = 0 and b* = 0, it was determined that the sample was gray. Delta E (ΔE) was a parameter that numerically determined the difference between two compared colors, most often the deviation of the color obtained in production from the standard.

The lightest ash was birch ash (L* = 43.9), the darkest oak ash (L* = 18.50), which had the opposite effect on the color of foams with these fillers (Table 4). The reddest a* color (positive) was contained in birch ash (2.6) and spruce ash (2.17), and green in paper ash (−1.25). Yellow was contained in all ashes except paper (the most in B and S’ ash); blue was characteristic of P ash.

#### 2.5.7. FTIR of Ashes

**Figure 5:**
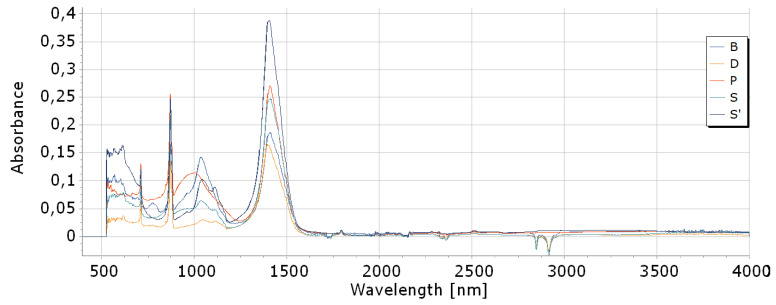
FTIR of ashes.

In the FTIR spectra of ashes, distinct bands with a maximum at about 1400 cm^−1^, at 1036 cm^−1^, at 872 cm^−1^, and at 712 cm^−1^ were recorded (Figure 5), but the band at about 1036 cm^−1^ was the largest and most distinct for birch ash. In the FTIR spectra of pine, oak, and birch, a band with a maximum at about 2920 cm^−1^ was also recorded. It was probably related to hydroxyl groups, -OH. These three ashes (S, D, B) also had a band at 2860 cm^−1^. It was probably related to the C-H bond. Spruce ashes and paper ashes did not have these two bands. They probably had lower hygroscopic properties. Data from Figure 5 were collected and compared in Table 5. Probably, the bands in the vicinity of 996–1057 cm^−1^ were related to vibrations of silicon–oxygen (Si-O) bonds, and in 1397–1410 cm^−1^ to vibrations of aluminum–oxygen (Al-O) bonds. The band at 873 cm^−1^ corresponded to carbonates, CO_3_^2−^ [66].

#### 2.5.8. DSC of Ashes

The literature reports that the ashes contained crystalline, amorphous, and glassy phases [67]. The main crystalline phase present in the product was calcium hydrogen phosphate dihydrate, CaHPO_4_ * 2H_2_O. With limited success, the amorphous phases of fly ash have been characterized using a number of analytical techniques, including polarized light microscopy, electron microscopy, X-ray diffraction, vibrational spectroscopy, gamma spectroscopy, nuclear magnetic resonance spectroscopy, thermal analysis, and selective dissolution [68,69,70]. The temperature properties of the ashes depended on the composition and properties of the ashes themselves [12], as well as on the type of material being burned (wood, coal, paper) [12,71,72,73].

**Figure 6:**
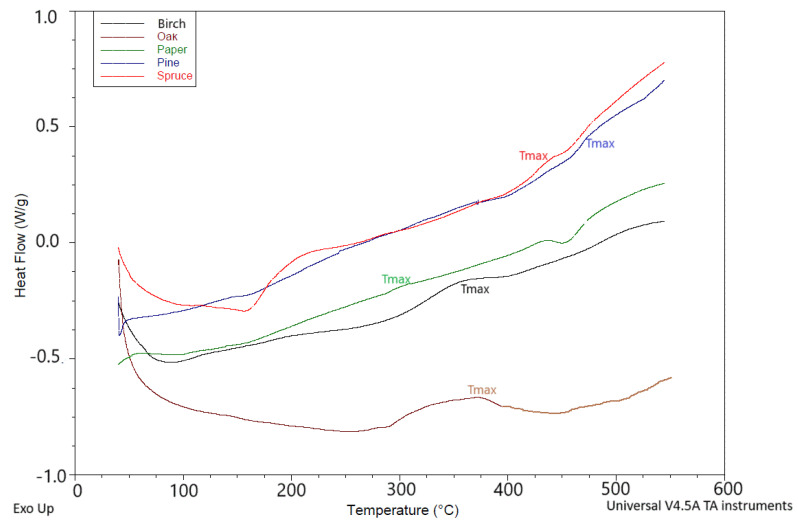
DSC of ashes.

The results of DSC studies of the ashes studied here are presented in Figure 6. The initial temperature of the exothermic peak (T_onset_), the temperature at which the maximum of the exothermic peak occurred (T_ma_x), and the final temperature of this peak (Tk) were determined—Table 6. In the DSC graph (Figure 3), peaks related to crystallization were observed at temperatures from 309 °C (paper) to 474 °C (pine). The lowest crystallization temperature was shown by paper ash (309 °C) and the highest by pine ash (474 °C).

#### 2.5.9. Analysis TGA of Ashes

**Figure 7:**
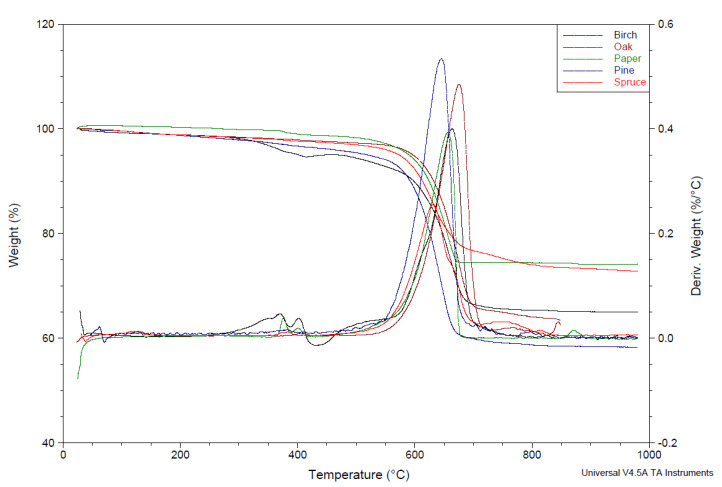
TGA and DTG of ashes.

Thermal properties of ashes were examined (Figure 7). Thermal analysis of ashes includes, among others, tests of mass change (thermogravimetry—TG, differential thermogravimetry—DTG) and temperature (differential thermal analysis—DTA). The results are presented in Table 7. The thermal stability and heat resistance of ashes are presented in Table 8. The beginning of mass change occurred the fastest for spruce ash (at 150 °C) and the latest for birch ash (at 280 °C). This means that oak ash needed the highest temperature to start its first mass loss. A very large residue after ash decomposition was observed at 1000 °C. It ranges from 63.5% (oak) to 72.8% (spruce). The highest T_max_. was characterized by oak ash (675 °C) and the lowest by spruce ash (630 °C).

### 2.6. Results and Discussion of Foams

#### 2.6.1. Foaming Process and Density

The directions of research on RPU result from their properties, such as density, water absorption, flammability, compressive strength, brittleness, thermal conductivity, or structure. It was noted that there is a close relationship between the properties of the obtained modified foams and their physical and chemical structure, as well as the type and amount of ash used. Determining individual times makes it possible to evaluate the course of the process and possibly modify it [43].

During the preparation of polyurethane foams, the times of subsequent stages of the foam formation process were determined. The processing parameters of the foams increased with the increasing content of the ash—Table 9. When foaming the foam composition with a specific concentration of the trimerization catalyst and urethane bond catalyst, the creaming time of all the compositions was 9–17 s.

In the case of adding ash to foams, a decrease in processing parameters (string gel time and tack free time or free rise time) was observed with the increase in the amount of these ashes in the foams. For example, the string gel time decreased from 31 s (W) to 19 s (PD and PB9). Only the addition of spruce ash caused all these parameters to increase.

The maximum reaction temperature (T_max_) ranged from 161 to 179 °C. No dependence of T_max_ on the amount of ash in the foam was observed. The type of ash also had no significance in terms of its effect on T_max_. The apparent density of foam was also examined (Table 9). The density of foams often determines the possibilities of their use. Other parameters, such as strength, water absorption, and cell size, also depend on it. Apparent density is a very important factor in the usability of RPUFs [74].

The density increased the fastest for foams with pine and oak ash, from about 40 Kg/m^3^ (W foam) to 65.37 Kg/m^3^ (PS9) or to 55.03 Kg/m^3^ (PD9)—Table 9. For other foams containing, e.g., birch ash (PB) or spruce ash (PS’), the density was similar to the density of W foam, e.g., for PB9 foam with maximum birch ash content it was 45.26 Kg/m^3^ and for PS’9 foam 43.59 Kg/m^3^. The density of foams with paper ash (PP) is different. With the addition of increasing amounts of P ash (from 1 wt.% to 9 wt.%), the density decreased slightly from about 40 Kg/m^3^ to about 37 Kg/m^3^. Foams of similar density were used in transport to provide protection for transported goods [74,75,76,77].

#### 2.6.2. Color of Foams

There was also measurement of the color of foam with 0 wt.% of ashes and with 7 wt.% and 9 wt.% content of ashes—Table 10 (description of colors—point 2.4.8.). Studies have shown that the lowest transparency (brightness), L* (amounting to 54.42), is characteristic of the PB9 foam, containing 9 wt.% birch ash. The highest L* transparency was characteristic of the PD9 foam with 9 wt.% oak ash (93.36). The reddest a* color (positive a* values) was contained in the foam with spruce ash (PS’9, 2.12) and birch ash (PB9, 0.37). The remaining foams were characterized by negative a* values, which indicated the content of green color (most in the W foam, −5.80). The yellow color (positive b*) characterized the W (0.25) and PB9 (3.39) foams, while the blue color (negative b*) occurred in the remaining foams. The lightest birch ash (B, Table 6) caused the PB series foams to darken from 61.09 (W) to 54.42 (PB9), and the darkest oak ash (D) brightened the PD series foams to 93.36 (PD9).

#### 2.6.3. Thermal Stability of Foams: Changes in Linear Dimensions, Volume, and Mass

Kuznia et al. found that magnesium oxide, which can cause an increase in the volume of samples, draws special attention [31]. They showed that the foams modified with ash were thermally stable. Fly ash inhibited thermal degradation of the material due to the thermal insulation effect of the gas trapped in the spherical ash particles [32].

In the case of testing our foams (PD, PS’, PP, PB, PS) modified with solid ash (D, S’, P, B, S), high thermal stability was observed. The type and amount of ash in the foams did not affect the change in the dimensions of the foam samples or the change in volume after 48 h of thermostating at 120 °C. The mass did not change either.

The results of the study show that ash can be successfully used as a modifier of thermal properties of polyurethane foams, improving the economics of production by reducing the density of the foam material and using a cheap filler.

#### 2.6.4. FTIR of Foams

FTIR was performed for the reference foam W and for foams containing 7 or 9 wt.% of individual ashes (PP9, PS’9, PD7, PS7, PB9). Interpretation of FTIR spectra was made on the basis of [78,79].

**Figure 8:**
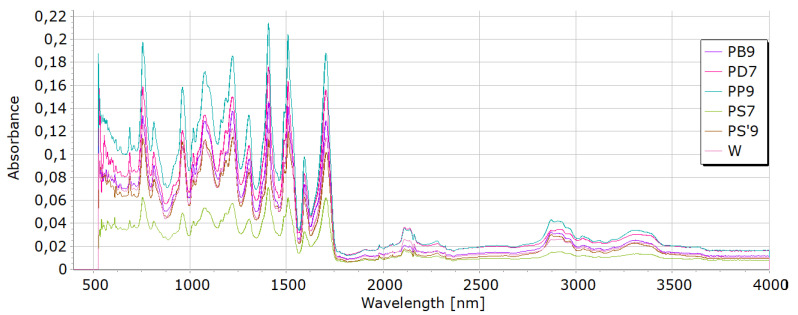
FTIR of foams with ashes: W—reference; PB9—with birch; PD7—witch oak; PS’9—with spruce; PS7—with pine; PP9—with paper.

FTIR analysis of foams (Figure 8) showed the presence of bonds, such as N–H (3325 cm^−1^, 1596 cm^−1^, 1512 cm^−1^), CH (2930 cm^−1^), –N=C=O (2276 cm^−1^), –N=C=N (2137 cm^−1^), C=O in a urethane bond (1713 cm^−1^) and isocyanurate ring (1411 cm^−1^), C–O (1076 cm^−1^), and –C=N in the trimer (1225 cm^−1^). These are bonds that occur in the structure of polyurethane–polyisocyanurate foam.

#### 2.6.5. Analysis TGA of Foams

**Figure 9:**
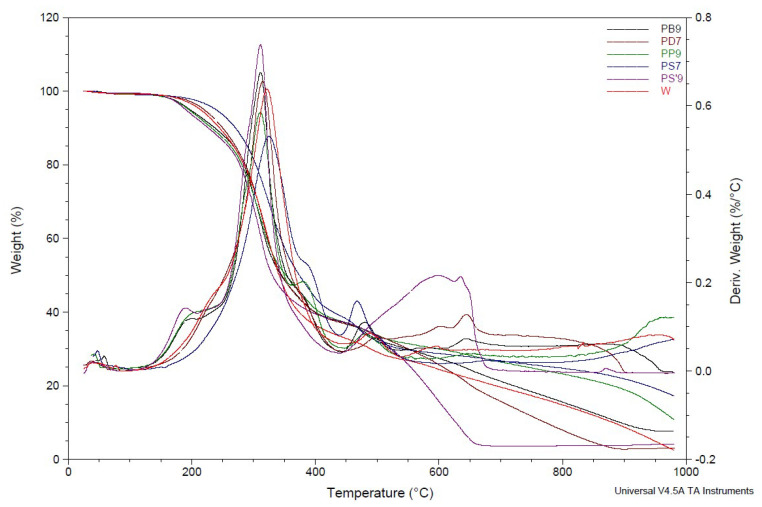
TGA and DTG of foams.

Thermal analysis of the foams included, among others, mass change studies (thermogravimetry—TG, differential thermogravimetry—DTG) and temperature (differential thermal analysis—DTA). The TGA curve (Figure 9) showed three stages of mass loss of the foams, the first to 100 °C, the second between 100 and 263 °C, and the third to 600 °C.

The beginning of the mass change, which is 0.5%, occurred at temperatures from 70 °C (foams W, PS7, PP9) to 87 °C (PD7—foam with 7 wt.% oak ash)—Table 11.

The decomposition of the foams began only at temperatures of about 255–271 °C, which indicated the excellent stability of the foams. The highest rate of mass loss of the foams began at a temperature of about 300 °C; for the foam with pine ash (PS7), it was even 312 °C—Table 11.

The residual coke content ranged from 3.1% (PD7) to 17% (PS7), and 5% for the foam W—Table 12. The residual mass index in the first stage was 0.5%, which was caused by the evaporation of free water and water bound in the foams. The residual mass index in the second stage was about 1%, which indicated that the degradation of the foam had begun. In the third stage, the degradation of the foam chains and the ashes contained in them occurred. The highest T_max_ was achieved by the foam with pine ash (324 °C) and was higher than the T_max_ of the ash-free foam (W, 321 °C)—Table 12. For the other foams, T_max_ ranged between 311 and 315 °C.

The test results showed that the most thermally stable foam was the one containing pine ash. The increased thermal stability of the foams was probably due to the formation of an ash layer that prevented decomposition.

#### 2.6.6. Flammability of Foams

Polyurethane foams are highly flammable due to the high content of oxygen, hydrogen, and carbon, and the presence of a large number of flammable hydrocarbon chain segments and porous structure [80]. Toxic fumes (CO, HCN, NO, etc.) and a large amount of heat are generated when they are combusted. They are a serious threat to the safety of human life and property [81]. Hot smoke from fires is harmful to both people and building materials. Four fire risk assessment indices are distinguished to examine the characteristics of smoke toxicity in materials. For measurement of the rate of gas production per unit of substance, apply the Toxic gas production index (ToxPI) [82]. Another smoke indicator is the smoke production index (TSPI). It is a logarithmic value of the amount of smoke produced by a material per unit of time. It reflects the total smoke emission per unit of surface area of this material.

**Figure 10:**
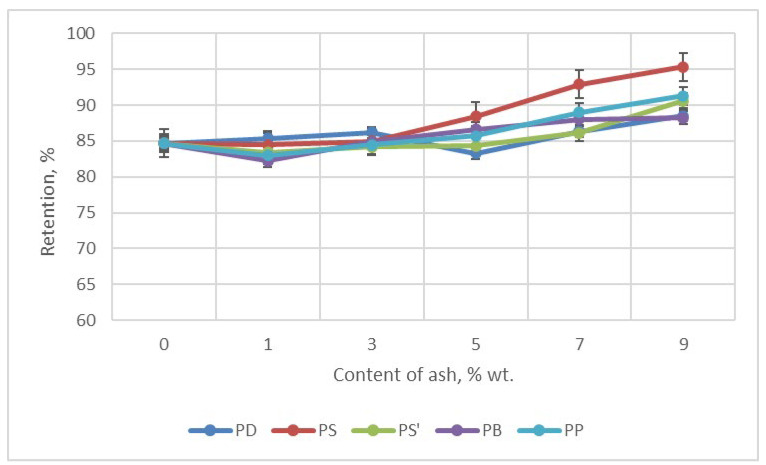
Retention of foam content ashes.

In the Butler vertical test, retention was determined—the residue after combustion of foams. The higher the retention, the less flammable the foam was. A clear improvement in foam retention is seen with the addition of at least 5 wt.% of each ash—Figure 10. Foam retention W was 84.70% (0 wt.% of ash). For foams with 9 wt.% of ash, retention ranged from 88.24% (PB9) to 95.31% (PS9).

Reducing the flammability of foams is extremely important when using them in construction, e.g., for insulation. Here, the determinant of reducing flammability was increasing the retention of foams. The reason for the increased retention was the addition of ash, which, by sticking to the foam cells, reduced the access of air to the interior of the cell, blocking the access of oxygen. In this way, it reduces the combustion of the foam material.

**Figure 11:**
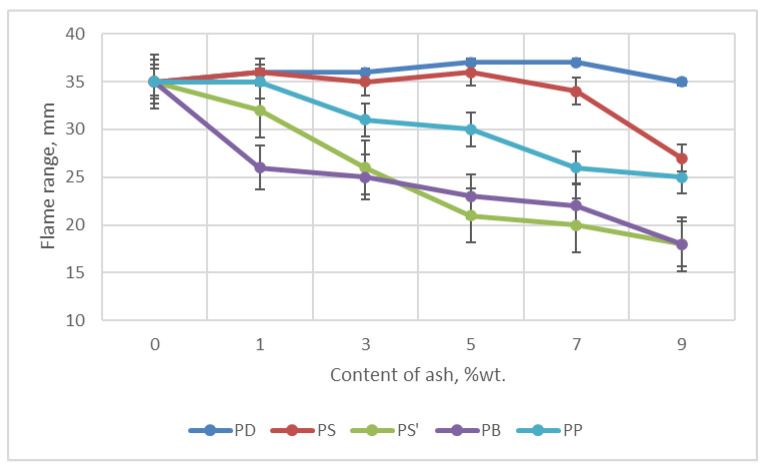
Dependence of flame range on ash content in foams.

In the horizontal test, the flame range was examined during 60 s of foam burning. This test also determined whether the foams were self-extinguishing after the flame was removed from the samples. The flame range of foams containing ash in the horizontal test decreased with the increase in ash content in the foam by up to 50% compared to the W foam containing no ash, e.g., for PS’ and PB foams from 36 mm to 18 mm—Figure 11. For the reference foam, it was 35 mm. The effect of ash on the reduction of foam flammability (including flame range) is clearly visible here. In the case of PS and PD foams, a slight increase in the flame range was observed with the addition of about 5 wt.% of ash. The foams were self-extinguishing. In general, the tests showed that the addition of ash reduced the degree of foam burning at the maximum (9 wt.%) ash content.

#### 2.6.7. Absorbability and Water Absorption of Foams

Absorbability (N) or water absorption (Ch) should depend on the density of the foams. The higher the density, the lower the N and Ch should be [83].

**Figure 12:**
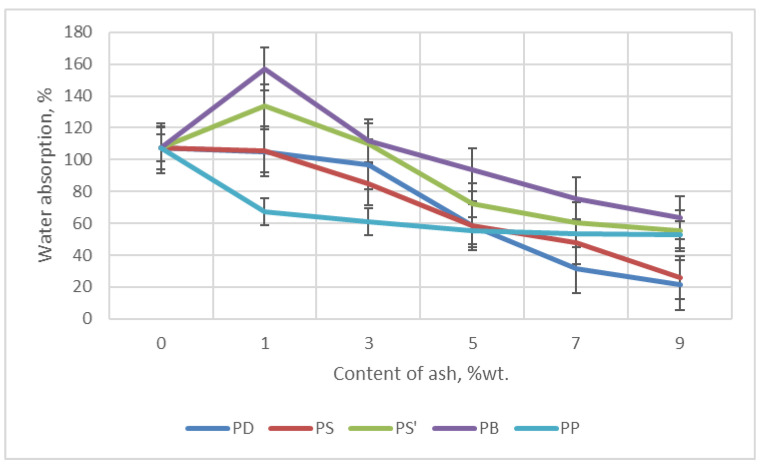
Dependence of water absorption on the content of ash in foam.

**Figure 13:**
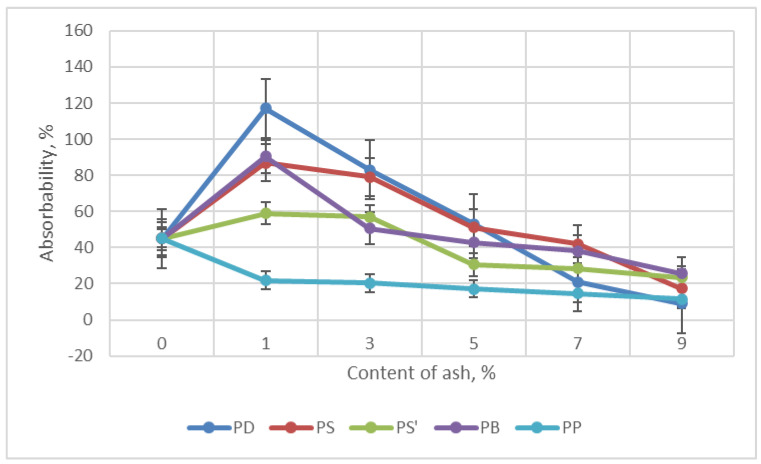
Dependence of absorbability on the content of ash in foam.

The content and type of ash significantly influenced the decrease in water absorption and absorbability of the foams tested here—Figure 12 and Figure 13. The greatest decrease in water absorption was observed for foams containing ash from oak, from 107.18% (foam W) to 21.15% (foam PD9). On the other hand, the lowest water absorption was characterized by foam containing 9% wt. ash from oak. This value decreased in relation to foam W from 37.56% (W foam) to 9.04% (PD9 foam).

The decrease in absorbability and water absorption of foams with ash content of 7–9 wt.% in foams can increase the frost resistance of foams when used in construction. On the other hand, the higher water absorption and water absorption for foams with ash content of 1–3 wt.% increases the interest in using foams by florists for flower arrangements.

#### 2.6.8. Content of Open and Closed Cells in Foams

The results of the closed-cell content were influenced by the material from which the foams were made, the type and amount of additive (if ash was added, for example), and the method of obtaining the foams.

**Figure 14:**
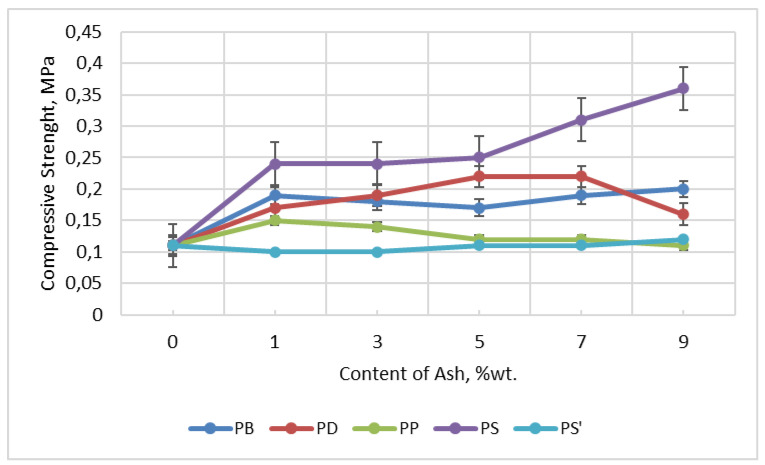
Dependence of compressive strength on ash content in foams.

The addition of ash to foams caused the cells to open. The closed-cell content of the foams tested here decreased from 97.18% (W foam) to 13.05% (PD9) and to 32.27% (PS13)—Table 13. In general, the strength of open-cell foams is lower than that of closed-cell foams. PS foams have fewer open cells (about 65%) and higher compressive strength (0.36 MPa, Figure 14), and PD foams have more open cells (about 85%) and three times lower compressive strength (0.15 MPa).

#### 2.6.9. Compressive Strength of Foams

**Figure 15:**
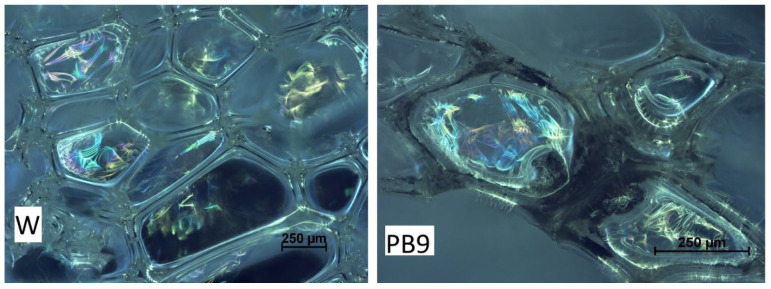
Foam structure.

Since fly ash improved mechanical properties and increased abrasion resistance [84], it was used as filler for polymers (polyethylene terephthalate, polypropylene, polyvinyl chloride). This had ecological (reduced the amount of waste) and economic (reduced the cost of products) significance. The beneficial effect of calcium contained in fly ash improved the mechanical properties of Portland cement (OPC), as demonstrated by Temuujin et al. [85] and Cho et al. [86]. The content of metals (metal oxides) in ashes and their beneficial effect on the strength of masonry mortars was also demonstrated by Iska-Kozak [87]. Materials PU used for wall insulation has usually approx. 0.15 MPa of compressive strength [64]. The strength test was carried out on five samples of each type. The result was averaged—Figure 14. The highest compressive strength was characteristic of foams containing pine ash. Addition of 9 wt.% pine ash to the foam caused a threefold increase in the compressive strength of the foams, from 0.11 MPa (W) to 0.36 MPa (PS9). Foam with paper and spruce ash has the lowest compressive strength, with values similar to the reference foam (0.11 MPa). Although the number of open cells increases with increasing ash content, the strength of the foams increases. This may be due to the stiffening of the structure by the ash and the strengthening of the cell skeletons. Ash, when mixed with liquid components used to create foam, creates a “concrete” shell, which is the reason for the increased compressive strength of the foams obtained here (Figure 15). The decrease in compressive strength in the case of PD9 foam was caused by the different cell sizes. It is likely that the addition of 9 wt.% oak ash requires more surfactant, which would cause foaming with a better (more uniform) structure.

#### 2.6.10. Foam Structure

The literature, e.g., [84,88,89], shows that fly ash improved mechanical properties and increased abrasion resistance. The results of our studies showed that the compressive strength of the foams increased (Section 2.6.10), in some cases the absorbability and water absorption decreased (Section 2.6.7), and the residue after combustion of the foams increased (Section 2.6.6). These properties resulted from the addition of tree and paper ash to the foams. The ash stuck to the structure of the foams (Figure 15), thus improving some of their properties. The photograph (Figure 15) shows the structure of W-foam without ash and an example foam with 9 wt.% birch ash content (PB9).

## 3. Conclusions

The addition of different types of ash to foams resulted in obtaining materials with different properties. Both the type and amount of ash in the foams influenced their properties. For example, pine ash caused a threefold increase in the compressive strength of the foams. Paper, spruce, and birch ash reduced the density of the foams, while oak and pine ash increased the apparent density. Adding smaller amounts of ash (1–3 wt.%) to foams slightly increased the absorbency and water absorption of the foams, while adding larger amounts of ash (5–9 wt.%) caused a significant decrease in the absorbency and water absorption of the foams. The ash caused that the foams were more thermally stable and had lower flammability. The strength of the foams was significantly influenced by their structure. The compressive strength of open-cell foams is three times lower than that of closed-cell foams, e.g., PS foams with about 65% open cells have a compressive strength of 0.36 MPa, and PD foams containing about 85% open cells have a compressive strength of 0.15 MPa. The foam material with ashes in the amount of up to 9% can be used in the packaging industry as spacers, protecting products against mechanical damage. The obtained foams can be used in transport to provide protection for transported goods. Water-absorbing foams can be used by florists for bouquets of live flowers.

## Figures and Tables

**Table 1:** Formulation of PU/PIR foams.

Filler (wt.%)					
Foam	Pine	Oak	Spruce	Birch	Paper
W	0	0	0	0	0
PS1	1	0	0	0	0
PS3	3	0	0	0	0
PS5	5	0	0	0	0
PS7	7	0	0	0	0
PS9	9	0	0	0	0
PD1	0	1	0	0	0
PD3	0	3	0	0	0
PD5	0	5	0	0	0
PD7	0	7	0	0	0
PD9	0	9	0	0	0
PS’1	0	0	1	0	0
PS’3	0	0	3	0	0
PS’5	0	0	5	0	0
PS’7	0	0	7	0	0
PS’9	0	0	9	0	0
PB1	0	0	0	1	0
PB3	0	0	0	3	0
PB5	0	0	0	5	0
PB7	0	0	0	7	0
PB9	0	0	0	9	0
PP1	0	0	0	0	1
PP3	0	0	0	0	3
PP5	0	0	0	0	5
PP7	0	0	0	0	7
PP9	0	0	0	0	9

**Table 2:** Results of ash density tests.

Ash	Skeletal Density (g/cm^3^) ± 0.0001	True Density (g/cm^3^) ± 0.0001	pH (-)
Paper P	2.9350	0.1472	9
Pine S	2.8749	0.2437	12
Spruce S’	2.9426	0.2108	12
Birch B	2.9031	0.2284	11
Oak D	2.9141	0.3133	13

**Table 3:** Results of SEM micrographs analysis of ashes.

Element Symbol	Weight					
	Conc. (wt.%)					
	ER *	P	S	S’	B	D
C	24.97	57.60	53.64	57.93	52.26	55.00
O	1.95	27.46	28.47	28.28	30.89	26.78
N	-	8.70	8.57	9.68	8.71	9.26
Ca	-	3.85	7.13	2.92	5.20	7.27
Si	-	1.01	0.53	0.22	1.00	0.26
Al	-	0.98	0.53	0.26	0.98	0.22
S	-	0.10	0.08	0.03	0.08	0.04
Mg	-	0.13	0.34	0.19	0.33	0.16
K	-	0.02	0.53	0.04	0.45	0.84
P	-	0.05	0.05	0.01	0.06	0.07
Na	-	0.01	0.13	0.06	0.06	0.00

* Epoxy resin.

**Table 4:** Values of color of ashes.

Sample	L* (-)	a* (-)	b* (-)	ΔE (-)
B	43.90	2.60	6.44	44.45
S	20.54	0.54	2.52	20.70
Ś	25.76	2.17	7.60	26.74
D	18.50	0.77	3.77	18.89
P	29.39	−1.25	−1.26	29.44

**Table 5:** Results of ash FTIR test.

Ash	Spectrum (cm^−1^)					
Paper—P	-	-	1410	996	873	712
Pine—S	2920	2860	1410	1055	873	712
Spruce—S’	-	-	1406	1057	873	714
Birch—B	2920	2860	1420	1036	874	712
Oak—D	2920	2860	1397	1055	873	712
Bond	-OH	C-H	Al-O	Si-O	Carbonates -CO_3_^2−^	

**Table 6:** Values of thermal transformations of ashes using the DSC.

Ash	Exothermic Behavior			
	T_onset_, (°C)	T_max_ (°C)	T_k_ (°C)	H (J/g)
Birch	303	362	412	7.7
Oak	290	372	412	19.2
Paper	286	309	322	2.3
Pine	463	474	487	6.8
Spruce	420	422	449	4.9

**Table 7:** Temperature of the beginning of mass change, beginning of decomposition, and greatest rate of mass loss of ashes.

Sample	Beginning of Mass Change		Beginning of Decomposition		Greatest Rate of Mass Loss	
	Temp. (°C)	Weight Loss (%)	Temp. (˚C)	Weight Loss (%)	Temp. (°C)	Weight Loss (%)
B	280	1.0	592	3.8	651	21
S	530	1.0	575	6.0	665	22
S’	150	1.0	540	4.0	630	16
D	187	1.0	575	4.0	670	17
P	225	1.0	608	2.0	625	12

**Table 8:** Temperature of 5%, 10%, and 20% ash mass loss and residue of ash at 1000 °C.

Sample	T_5%_ (°C)	T_10%_ (°C)	T_20%_ (°C)	T_max_. (°C)	Residue at Temp. 1000 °C (%)
B	402	592	648	664	65.0
S	530	590	625	665	62.0
S’	573	612	659	632	72.8
D	597	632	663	675	63.5
P	590	620	655	656	74.2

**Table 9:** Processing times, maximum reaction temperatures (T_max_), and apparent density of PU/PIR foams.

Foam	Cream Time (s)	String Gel Time (s)	Tack Free Time (s)	Free Rise Time (s)	T_max_ (°C)	Apparent Density (Kg/m^3^)
W	10	31	28	40	170.6	40.49 ± 3.6
PS1	13	29	42	42	173.0	49.70 ± 3.6
PS3	13	33	33	35	177.0	53.13 ± 3.6
PS5	13	29	29	29	176.0	58.18 ± 3.6
PS7	13	28	29	30	176.0	64.82 ± 3.6
PS9	13	26	27	28	179.0	65.37 ± 3.6
PB1	9	20	28	38	173.0	35.32 ± 3.6
PB3	9	22	23	38	172.1	36.52 ± 3.6
PB5	9	24	26	37	169.7	41.37 ± 3.6
PB7	9	21	26	37	169.0	44.03 ± 3.6
PB9	9	19	24	36	161.0	45.26 ± 3.6
PS’1	10	20	24	31	169.7	32.80 ± 3.6
PS’3	10	20	24	33	168.2	36.09 ± 3.6
PS’5	10	20	24	35	172.1	37.49 ± 3.6
PS’7	16	40	42	45	171.0	38.70 ± 3.6
PS’9	17	42	45	48	168.3	43.51 ± 3.6
PP1	9	26	31	39	169.6	40.24 ± 3.6
PP3	9	25	31	39	169.9	42.63 ± 3.6
PP5	9	23	31	37	169.8	37.38 ± 3.6
PP7	9	25	30	37	169.7	36.22 ± 3.6
PP9	9	24	27	36	167.8	37.36 ± 3.6
PD1	13	23	25	32	178.9	38.53 ± 3.6
PD3	12	21	23	32	186.2	43.51 ± 3.6
PD5	12	21	23	30	177.3	44.56 ± 3.6
PD7	12	20	21	29	173.0	51.68 ± 3.6
PD9	12	19	21	26	172.1	55.03 ± 3.6

**Table 10:** Values of color of foams.

Sample	L* (-)	a* (-)	b* (-)	ΔE (-)
W	61.09	−5.80	0.25	61.37
PB9	54.42	0.37	3.39	54.53
PS7	73.03	−0.63	−1.89	74.10
PS’9	68.48	2.12	−3.03	68.58
PD7	93.36	−0.63	−1.89	93.38
PP9	79.99	−2.54	−2.54	80.07

**Table 11:** Temperature of the beginning of mass change, beginning of decomposition, and the highest rate of mass loss of PU/PIR foams.

Sample	Beginning of Mass Change		Beginning of Decomposition		Highest Rate of Mass Loss	
	Temperature (˚C)	Weight Loss (%)	Temperature (°C)	Weight Loss (%)	Temperature (°C)	Weight Loss (%)
W	70	0.5	204	4.0	300	28
PB9	78	0.5	235	10.0	300	28
PS7	70	0.5	199	2.5	312	25
PS’9	80	0.5	202	7.0	300	27
PD7	87	0.5	205	4.0	285	26
PP9	70	0.5	201	3.5	300	26

**Table 12:** Temperature of 5%, 10%, 20%, and 50% foam mass loss and residue of foam at 1000 °C.

Sample	T_5%_ (°C)	T_10%_ (°C)	T_20%_ (°C)	T_50%_ (°C)	Residue at Temp. 1000 °C (%)	T_max_ (°C)
W	214	235	284	345	5.0	321
PB9	197	235	280	344	8.0	312
PS7	239	275	304	374	17.2	324
PS’9	189	225	280	330	5.0	311
PD7	220	250	287	346	3.1	315
PP9	195	250	281	351	10.8	311

**Table 13:** Results of testing the content of open and closed cells.

Foam	Open Cells (%)	Closed Cells + Skeleton (%)
W	2.88 ± 0.01	97.18 ± 0.01
PS5	64.50 ± 0.01	35.50 ± 0.01
PS9	67.73 ± 0.01	32.27 ± 0.01
PD5PD9	81.85 ± 0.01 86.95 ± 0.01	18.15 ± 0.01 13.05 ± 0.01

## Data Availability

The original contributions presented in this study are included in the article. Further inquiries can be directed to the corresponding author.
